# Identification and Characterization of Cefotaxime Resistant Bacteria in Beef Cattle

**DOI:** 10.1371/journal.pone.0163279

**Published:** 2016-09-19

**Authors:** Raies A. Mir, Thomas A. Weppelmann, Judith A. Johnson, Douglas Archer, J. Glenn Morris, KwangCheol Casey Jeong

**Affiliations:** 1 Department of Animal Sciences, Institute of Food and Agricultural Sciences, University of Florida, Gainesville, Florida, United States of America; 2 Emerging Pathogens Institute, University of Florida, Gainesville, Florida, United States of America; 3 Department of Environmental and Global Health, College of Public Health and Health Professions, University of Florida, Gainesville, FL, United States of America; 4 Department of Pathology, Immunology and Laboratory Medicine, College of Medicine, University of Florida, Gainesville, FL, United States of America; 5 Food Science and Human Nutrition Department, Institute of Food and Agricultural Sciences, University of Florida, Gainesville, Florida, United States of America; Cornell University, UNITED STATES

## Abstract

Third-generation cephalosporins are an important class of antibiotics that are widely used in treatment of serious Gram-negative bacterial infections. In this study, we report the isolation of bacteria resistant to the third-generation cephalosporin cefotaxime from cattle with no previous cefotaxime antibiotic exposure. The prevalence of cefotaxime-resistant bacteria was examined by a combination of culture based and molecular typing methods in beef cattle (n = 1341) from 8 herds located in North Central Florida. The overall prevalence of cefotaxime-resistant bacteria was 15.8% (95% CI: 13.9, 17.8), varied between farms, and ranged from 5.2% to 100%. A subset of isolates (n = 23) was further characterized for the cefotaxime minimum inhibitory concentration (MIC) and antibiotic susceptibility against 10 different antibiotics, sequencing of nine β- lactamase genes, and species identification by 16S rRNA sequencing. Most of the bacterial isolates were resistant to cefotaxime (concentrations, > 64 μg/mL) and showed high levels of multi-drug resistance. Full length 16S rRNA sequences (~1300 bp) revealed that most of the isolates were not primary human or animal pathogens; rather were more typical of commensal, soil, or other environmental origin. Six extended spectrum β-lactamase (ESBL) genes identical to those in clinical human isolates were identified. Our study highlights the potential for carriage of cefotaxime resistance (including “human” ESBL genes) by the bacterial flora of food animals with no history of cefotaxime antibiotic exposure. A better understanding of the origin and transmission of resistance genes in these pre-harvest settings will be critical to development of strategies to prevent the spread of antimicrobial resistant microorganisms to hospitals and communities.

## Introduction

The emergence of bacterial pathogens resistant to treatment with antibiotics is one of the most critical challenges to modern medicine and represents a major threat to public health. Our ability to combat the rise in infections caused by antimicrobial resistant microorganisms (ARMs) will be benefited by understanding of both the sources of ARMs in the environment and the mechanisms of antibiotic resistance. It is becoming more evident that the natural bacterial resistome plays an important role in the evolution and spread of resistance [1,2] with extensive use of antibiotics in food animal production likely accelerating the acquisition of antimicrobial resistance by human pathogens [3].

Third generation cephalosporins are widely used for the treatment and prevention of bacterial infections in hospitals, but not in food animal production for the prevention purpose since 2012 [4,5]. Bacterial resistance to third generation cephalosporins is often conferred by production of extended spectrum β-lactamase (ESBL) enzymes [6]. While the presence of ESBL producing bacteria has also been reported in food animals exposed to antibiotics [6–11], currently there is little data regarding the prevalence of ARMs in animals raised without certain antibiotics such as cefotaxime. To investigate the phenomenon of antibiotic resistance occurrence, we examined the prevalence of bacteria resistant to cefotaxime from cattle with no previous cefotaxime exposure at three farms in North Central Florida. The majority of the isolates were commensal bacteria commonly found in nature; however they were frequently multi-drug resistant and possessed ESBL genes identical to isolates from hospital and community acquired infections.

## Materials and Methods

### Ethics statement

Standard practices of animal care and use were applied to animals used in this project. The research protocols used in this study were approved by the University of Florida Institutional Animal Care and Use Committee (IACUC Protocol #: 201308027).

### Study location and sample collection

A total of 1,341 fecal samples were collected from cattle belonging to 8 different herds housed at three different farms from the North Central Florida. The herds were located at the Beef Research Unit (BRU) in Waldo, the North Florida Research and Education Center (NFREC) in Marianna, and a feedlot operation in Suwanee. Access to the farms to collect fecal samples was permitted by Mr. J. Danny Driver (manager of BRU), Dr. G. Cliff Lamb (Associate Center Director of NFREC), and the owner of Suwanee Feedlot. The farms are within approximately 300 miles from one another and distributed throughout Northern and North Central Florida. During the study period, the cattle were not transported between farms or different herds in this study. At NFREC, female cattle aged between 2 to 8 years were sampled in September and October of 2013; at BRU and the feedlot, both male and female cattle aged between 1 and 2 years were sampled in March and July, respectively. Sterile cotton swabs were used to collect fecal samples directly from the recto-anal junction (RAJ) of each animal with no previous cefotaxime exposure. Following sample collection, fecal swabs were placed and transported in 15 ml conical tubes on ice to stop bacterial growth to the Emerging Pathogens Institute at the University of Florida, and processed the same day. Cefotaxime resistant bacteria were isolated and characterized using the following methods.

### Isolation of cefotaxime resistant bacteria

Samples were serially diluted (up to 10^−4^) with Luria Bertani (LB) broth and then plated on Tryptic Soy or MacConkey agar (BD, USA) containing lactose as carbon source and cefotaxime (4 μg/mL). Plates were incubated at 37°C and examined after 24 hours for the enumeration of bacterial colonies. Resistance to cefotaxime due to the production of extended spectrum β- lactamase was identified by streaking cefotaxime resistant isolates on ChromAgar ESBL (CHROMagar, France) as previously described [12,13]. Four colonies from each fecal sample with the presence of cefotaxime resistant bacteria were purified. A total of 1,200 colonies were further subjected to minimum inhibitory concentration (MIC) test against cefotaxime using microbroth dilution method and following the CLSI guidelines. KCJ1409, an ESBL producing human clinical isolate, was included in MIC test as a positive control. Twenty-three colonies including KCJ1409 were selected with MIC of cefotaxime greater than 16 μg/mL and tested for the presence of *bla*-TEM and *bla*-CTX-M genes by PCR using primers as described below (Table 1).

**Table 1:** Primers used to amplify ESBL and 16S rRNA genes.

Target genes	Primer name	Primer sequence	Amplicon size (nt)	References
*bla*TEM-F	KCP 550	ATGAGTATTCAACAT TTC CG	840	[14]
*bla*TEM-R	KCP 551	CCAATGCTTAATCAG TGA GG		
*bla*SHV-F	KCP 552	TTCGCCTGTGTATTATCTCCCTG	854	[14]
*bla*SHV-R	KCP 553	TTAGCGTTGCCAGTGYTCG		
*bla*CMY-F	KCP 556	ATGATGAAAAAATCGTTATGC	1200	[14]
*bla*CMY-R	KCP 557	TTGCAGCTTTTCAAGAATGCGC		
*bla*OXA-1-F	KCP 558	ATGAAAAACACAATACATATCAACTTCGC	820	[14]
*bla*OXA-1-R	KCP 559	GTGTGTTTAGAATGGTGATCGCATT		
*bla*OXA-2-F	KCP 560	ACGATAGTTGTGGCAGACGAAC	602	[14]
*bla*OXA-2-R	KCP 561	ATYCTGTTTGGCGTATCRATATTC		
*bla*ACC-like-F	KCP 562	AGCCTCAGCAGCCGGTTAC	818	[14]
*bla*ACC-like-R	KCP 563	GAAGCCGTTAGTTGATCCGG		
*bla*VEB-F	KCP 564	ATTTAACCAGATAGGACTACA	1000	[14]
*bla*VEB-R	KCP 565	CGGTTTGGGCTATGGGCAG		
*bla*DHA con-F	KCP 566	TGATGGCACAGCAGGATATTC	997	[14]
*bla*DHA con-R	KCP 567	GCTTTGACTCTTTCGGTATTCG		
*bla*CTXM pan-F	KCP 685	TTTGCGATGTGCAGTACCAGTAA	500	[14]
*bla*CTXM pan-R	KCP 686	CGATATCGTTGGTGGTGCCATA		
16S rRNA-F	KCP 812	CAG GCC TAA CAC ATG CAA GTC	1300	[15]
16Sr RNA-R	KCP 813	GGG CGG WGT GTA CAA GGC		

The twenty-three bacteria were further tested for susceptibility to ten different antimicrobial compounds according to the Clinical and Laboratory Standards Institute [16]. Briefly, the isolates were tested using the standard Kirby Bauer disk diffusion method on Mueller Hinton agar to generate an antibiogram of the cefotaxime resistant isolates. The control strains used for the antibiotic susceptibility test were *Escherichia coli* (ATCC 35401), *Staphylococcus aureus* (ATCC 25923) *and Pseudomonas aeruginosa* (ATCC 27853). The following antimicrobial disk concentrations were used: Ampicillin (A; 10μg), Cefotaxime (X; 30 μg), Ceftazidime (Z; 30 μg), Ceftriaxone (R; 30 μg), Chloramphenicol (C; 30 μg), Ciprofloxacin (I; 5 μg), Gentamicin (G; 10 μg), Nalidixic acid (N; 30 μg), Streptomycin (S; 10 μg), and Tetracycline (T; 30 μg) (BD, USA).

#### Characterization of cefotaxime resistant bacteria

The 23 cefotaxime resistant bacteria were analyzed for the presence of nine different ESBL genes (Table 1) and taxonomic identification was conducted at the species level. Genomic DNA was extracted with a Qiagen DNA mini kit and used as a template for multiplex polymerase chain reaction (PCR) to amplify nine ESBL genes [14] and 16S rRNA gene [15] using the primer sets shown in (Table 1). The PCR conditions for all reactions were: 95°C for 5 minutes for initial denaturation, 30 cycles of 95°C for 30 seconds, 55°C for 35 seconds, 72°C for 90 seconds, and a final extension at 72°C for 7 minutes. All products were resolved on 1% agarose gel stained with ethidium bromide and visualized with a UV gel doc system (Bio-Rad, USA). The PCR products from the most frequently observed genes *bla*-TEM, *bla*-CTX-M, and the 16SrRNA were eluted using QIAEX II Gel Extraction Kit (Qiagen Inc, Germany) and sequenced by the Interdisciplinary Center for Biotechnology Research (ICBR) at University of Florida. The online NCBI nucleotide BLAST program was used to compare the homology of the sequences from the isolates with ESBL genes and 16S rRNA sequences of other organisms [17]. The sequences of the ESBL genes and 16S rRNA from the isolates were aligned and a maxium likelihood tree was constructed using the Jukes and Cantor model in MEGA version 6.0 software with 1000 bootstrap replications with a bootstrap value of 0.95 (95%) [18]. Tree annotations were performed using FigTree (version 1.4.2.).

## Results

### Isolation of cefotaxime resistant bacteria in cattle

**Fig 1:**
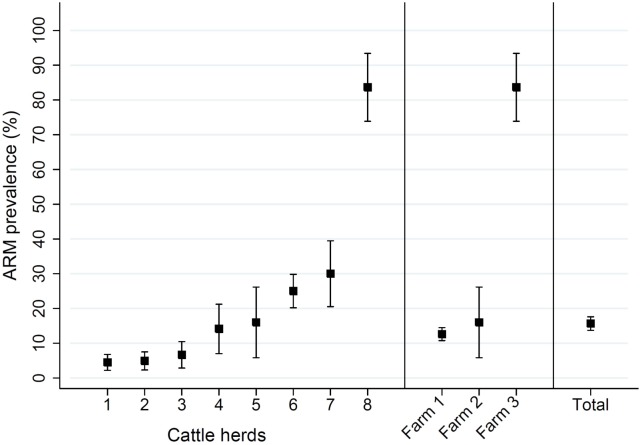
Cefotaxime resistance in cattle. The prevalence of cefotaxime resistant bacteria isolated from eight cattle herds housed at three farms located in North-Central Florida is presented with 95% confidence intervals, along with the total prevalence. Farm 1 includes herds of 1, 2, 3, 4, 6, and 7. Farm 2 and 3 include herd 5 and 8, respectively.

The detection rate of cefotaxime resistant bacteria in samples collected from all of the cattle herds was 15.7% (95% CI: 13.7%, 17.6%) and ranged in individual herds from 4.5% to 83.6% (Fig 1, Table 2). The prevalence of cattle shedding with cefotaxime resistant bacteria on the two farms (NFREC vs. BRU) raising calves and cattle with a loose system of pasture-grazing was not significantly different (*P* = 0.99, Student’s T-test), however both had a significantly lower (*P* < 0.001) prevalence of cefotaxime resistant bacteria than the more intensive feedlot operation (12.8% or 16.0% vs 83.6%).

**Table 2:** Prevalence of cefotaxime resistant bacteria from different cattle herds.

Cattle herd	Geographic Location†	Samples (n)	Number Cef ^R^^††^	Prevalence (%)	95% Confidence Interval	
1	NFREC, Marianna, Fl	312	14	4.5	2.2	6.8
2	NFREC, Marianna, Fl	265	13	4.9	2.3	7.5
3	NFREC, Marianna, Fl	165	11	6.7	2.9	10.5
4	NFREC, Marianna, Fl	92	13	14.1	7.0	21.2
5	BRU, Waldo, Fl	50	8	16.0	5.8	26.2
6	NFREC, Marianna, Fl	312	78	25.0	20.2	29.8
7	NFREC, Marianna, Fl	90	27	30.0	20.5	39.5
8	Feedlot, Suwannee, Fl	55	46	83.6	73.9	93.4
Total	North Central Florida	1341	210	15.7	13.7	17.6

^†^ Location abbreviations: North Florida Research and Education Center (NFREC) and Beef Research Unit (BRU)

^††^ Number of cattle with bacteria isolated on MacConkey agar with 4 ug/mL cefotaxime

**Fig 2:**
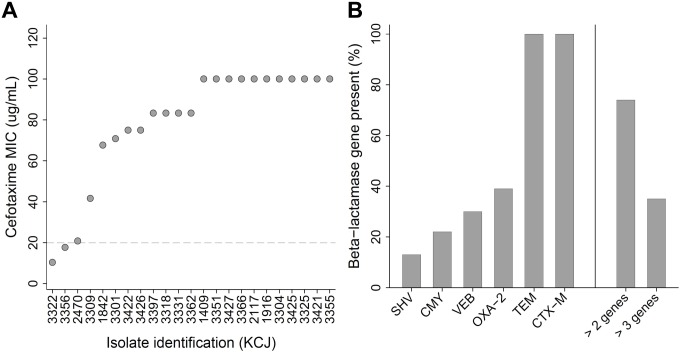
MIC of cefotaxime and multiplex PCR for ESBL genes. (A) The results of the subset of cefotaxime resistant bacterial isolates subjected to minimum inhibitory concentration MIC testing with cefotaxime. (B) The percentages (%) of isolates that had the presence of nine different β-lactamase genes determined by polymerase chain reaction PCR; only six genes were identified, with isolates frequently carrying more than one β-lactamase gene.

**Fig 3:**
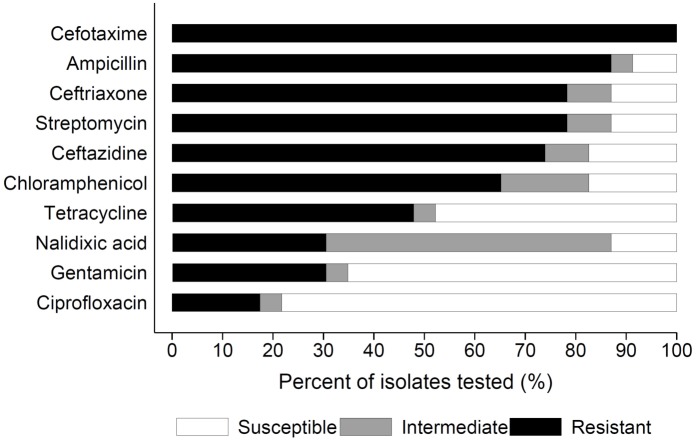
Antimicrobial Susceptibility Test. The antibiotic susceptibility test results of the 23 cefotaxime resistant bacterial isolates toward ten different antimicrobial compounds are presented as the percentage (%) of isolates that exhibited complete resistance black, intermediate resistance gray, and no resistance white to the antibiotics listed on the y axis.

Over 90% of the subset of bacterial isolates tested had a minimum inhibitory concentration (MIC) of cefotaxime greater than or equal to 16 μg/mL, with approximately half (48%) having an MIC greater than or equal to 100 μg/mL of cefotaxime (Fig 2A). Of the nine ESBL genes commonly reported in human clinical isolates, *bla* CTX-M was present in all of the cefotaxime resistant isolates; *bla* CMY consensus was present in 22%, *bla* SHV in 13%, *bla* OXA*-2* in 39%, and *bla* VEB consensus in 30% (Fig 2B). No amplification of *bla* ACC, *bla* OXA-1, or *bla* DHA consensus genes was detected in any of the isolates. In addition, *bla* TEM, which is not considered an ESBL, was present in all of the cefotaxime resistant isolates. Over 70% of the isolates were carrying more than two ESBL genes and 35% carried more than three ESBL genes. Antibiotic susceptibility testing on the cefotaxime resistant isolates revealed high levels of resistance to several β-lactam antibiotics including ampicillin (87%), other cephalosporins such as ceftriaxone (78%) and ceftazidime (74%), streptomycin (78%), and chloramphenicol (65%) (Fig 3; Table 3). A lower percentage of the cefotaxime resistant isolates were highly resistant to Tetracycline (48%), Nalidixic acid (30%), Gentamicin (30%), and Ciprofloxacin (17%). Besides individual antibiotics, the bacterial isolates tested were also frequently multi-drug resistant, with resistance to 5 (18%), 6 (45%), 7 (35%), and 8 or more (2%) different antibiotic classes, respectively.

**Table 3:** Antibiotic susceptibility testing and ChromAgar ESBL characterization.

Strain ID	Strains	AST Profile†	ChromAgar ESBL
1409	*Escherichia coli*	AXRCINST	Deep purple
1842	*Acinetobacter calcoaceticus*	AX	Metallic blue
1916	*Ochrobactrum anthropi*	AXZRGST	Yellow
2117	*Ochrobactrum intermedium*	ACX	Yellow
2470	*Escherichia coli*	AXZC	Deep purple
3301	*Escherichia coli*	XGS	Deep purple
3304	*Acinetobacter calcoaceticus*	AXZRCS	Metallic blue
3309	*Escherichia coli*	AXZRCGST	Deep purple
3318	*Ochrobactrum intermedium*	AXZRCS	Yellow
3322	*Agrobacterium tumefaciens*	XZ	Metallic blue
3325	*Pseudomonas plecoglossicida*	AXZRCST	Yellow
3331	*Ochrobactrum anthropi*	AXZRG	Yellow
3351	*Ochrobactrum anthropi*	AXZRCNS	Yellow
3355	*Ochrobactrum intermedium*	AXZRCIGNST	Yellow
3356	*Escherichia coli*	AXZRCS	Deep purple
3362	*Ochrobactrum intermedium*	AXZRCS	Yellow
3366	*Ochrobactrum intermedium*	AXZRCST	Yellow
3397	*Ochrobactrum intermedium*	AXZRCIGNST	Yellow
3421	*Ochrobactrum intermedium*	XNS	Yellow
3422	*Pseudomonas plecoglossicida*	XCN	Yellow
3425	*Ochrobactrum intermedium*	AXZRC	Yellow
3426	*Escherichia coli*	AXZRCIGNST	Deep purple
3427	*Ochrobactrum anthropi*	AXZRST	Yellow

^†^ Antibiotic abbreviations: Ampicillin (A), Cefotaxime (X), Ceftazidime (Z), Ceftriaxone (R), Chloramphenicol (C), Ciprofloxacin (I), Gentamicin (G), Nalidixic acid (N), Streptomycin (S), and Tetracycline (T)

### Genetic characterization of a subset of cefotaxime resistant isolates

After streaking a subset of cefotaxime resistant isolates on selective ChromAgar ESBL media, 61% produced yellow colonies, 26% produced purple colonies, and 13% produced metallic blue colonies; indicating different bacterial genera were responsible for ESBL enzyme production (Table 3).

**Fig 4:**
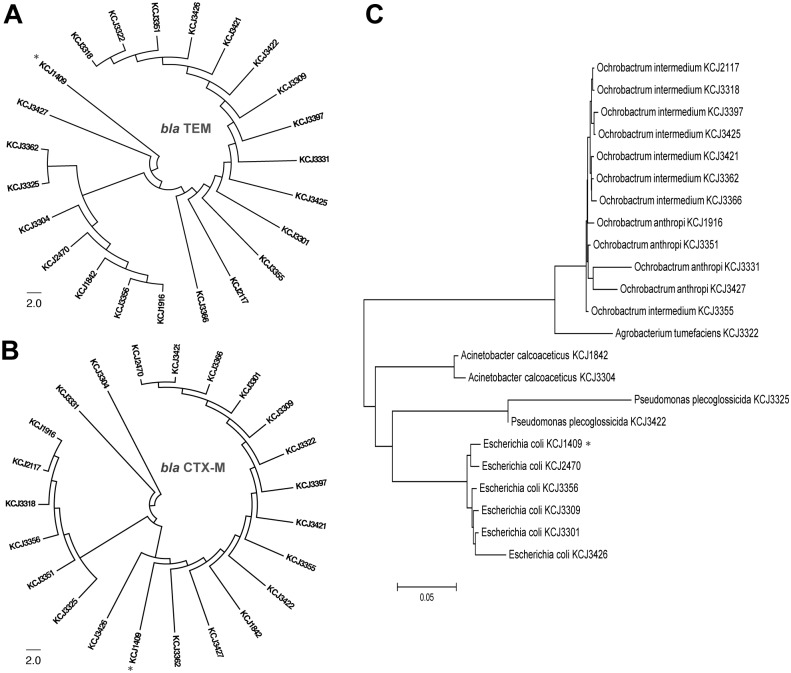
Caption:Phylogenetic analysis of the resistance genes. (A and B) Neighbor-joining trees from the phylogenetic analysis of the 23 isolates that contained the β-lactamase genes. *bla* TEM (A) and *bla* CTX-M (B) demonstrate the similarity of the β-lactamase genes to clinical isolates from human infections. (C) The sequences of the 16S ribosomal RNA from the same isolates were used to identify the genus and species of the cefotaxime resistant bacteria and show their sequence similarity. Asterisks indicate a human clinical isolate KCJ1409.

Since the *bla* TEM and *bla* CTX-M genes were found in all of the cefotaxime resistant isolates tested, the sequence similarity was compared to known isolates in the NCBI database. A Nucleotide Blast (BLASTN) search of the 23 *bla* TEM positive isolates indicated high similarity (> 98% identity) with previously reported *bla* TEM genes identified in human infections from hospitals and community-acquired infections. A BLASTN search of the 23 *bla* CTX-M positive isolates also indicated high similarity to previously reported *bla* CTX-M-15 (> 99% identity) and *bla* CTX-M-1 (> 98% identity). Two animal isolates, KCJ3331 and KCJ3304, and a human clinical isolate KCJ1409 encoded CTX-M-1, while others encoded CTX-M-15. The sequences were subjected to further analyses to determine the genetic relatedness between the *bla* TEM and *bla* CTX-M genes identified in the 23 cefotaxime resistant isolates. The neighbor-joining phylogenetic trees for both *bla* TEM and *bla* CTX-M genes presented in Fig 4A and 4B, respectively, shows that several isolates were grouped together with the human clinical isolate KCJ1409. A homology search of 16S rRNA gene sequences using the NCBI database was used to identify cefotaxime resistant isolates, which included commensal, soil and plant bacteria that commonly occur in the environment. Bacterial species listed in the order of isolation frequency were: *Ochrobactrum intermedium* (34.8%), *Escherichia coli* (26.1%), *Ochrobactrum anthropi* (17.4%) *Acinetobacter calcoaceticus* (8.7%), *Pseudomonas plecoglossicida* (8.7%), and *Agrobacterium tumefaciens* (4.3%). The phylogenetic relationship between these bacterial isolates reveals genetic diversity within each species (Fig 4C).

## Discussion

In the current study, beef cattle with no previous cefotaxime antibiotic exposure were colonized by bacteria resistant to the third generation cephalosporin cefotaxime. Antibiotic-producing microorganisms occur regularly in soil, on plants, and aquatic environments [19,20] and large-scale mixing of these organisms with bacteria in the environment can facilitate evolution and dissemination of the natural antibiotic resistome [20,21]; however the finding of bacteria resistant to semi-synthetic antibiotics such as cefotaxime in food animals with no previous exposure is surprising. Since environmental bacteria or commensal species in food animals have the potential for transfer of antimicrobial resistance genes to clinically relevant human pathogens [22], the natural occurrence of ARMs represents a potential public health concern [23].

Food animal production (including beef feedlot production) in North America is becoming progressively more intensive and density of the animals housed in feedlot operations are much greater than that of animals housed on cow-calf operations [24]. All of the farms included in this study have loose housing management systems of animals except herd 7 (animals were housed in a feeding efficiency facility mimicking a feedlot) and herd 8, which are intensive feedlots. Since these animals had a significantly higher prevalence of ARMs and feedlots are the final stage of food animal production prior to meat processing and distribution, these animals represent a potential source of ARM entry to the community. Furthermore, the majority of bacteria isolated from cattle subjected to minimum inhibitory concentrations (MIC) of cefotaxime revealed clinically relevant levels of antibiotic resistance (≥ 64μg/ml of cefotaxime) [16]. Given the high MIC of these isolates, it would be extremely difficult to treat infections caused these bacteria or infections resulting from horizontal gene transfer to clinical pathogens [25,26].

Along with the high MIC of cefotaxime, the results from the multiplex PCR for ESBL genes indicated that the subsample of bacteria isolated from cattle likely carried multiple genes conferring resistance to other β-lactam antibiotics (Fig 2). Six of the nine different ESBL genes were identified with the majority of isolates positive for more than one gene and all of the cefotaxime resistant bacteria isolated from cattle were resistant to multiple antibiotics (Fig 3, Table 3), suggesting a variety of ESBL genes are prevalent in animal farms. The ESBL gene *bla* CTX-M were found in 100% of the farm isolates and had sequences with a high level of genetic similarity to clinically relevant pathogens isolated from hospitals [22], suggesting that *bla*-CTX-M might be transmitted to the animal farms from human hospitals through the environments. Similarly, results from the 16S rRNA sequencing revealed the presence of antibiotic resistance genes in multiple bacterial genera, which could increase the likelihood of a spillover to clinically relevant pathogens [27]. Members of *Enterobacteriaceae* have frequently been implicated in multi-drug resistant human infections [14,28,29] while environmental bacteria including *Agrobacterium tumefaciens*, *Acinetobacter* sp., and *Ochrobactrum* sp. have been less frequently associated with antibiotic resistant human infections [30–31]. However, as shown in this study, the emerging opportunistic human pathogens have already acquired multi-drug resistance, suggesting they may cause serious health problems as *Enterobacteriaceae*.

Our study highlights the potential for carriage of cefotaxime resistance in food animals. Cefotaxime resistance was the focus of this study because cephalosporins are widely used in human medicine; however it became evident that these isolates also possessed high levels of resistance against other clinically relevant antibiotics. Given the antibiotic resistance profiles of these environmental isolates as well as their genetic similarity to human clinical isolates, we speculate that if these resistance genes were transferred to communities, hospitals, or directly to pathogens, the resulting infections could represent a substantial threat to public health. Increased understanding of natural antibiotic resistance at the pre-harvest stage of food animal production will result in the development of strategies to prevent the spread of ARMs to hospitals and communities.
